# Potential Role for Osteocalcin in the Development of Atherosclerosis and Blood Vessel Disease

**DOI:** 10.3390/nu10101426

**Published:** 2018-10-04

**Authors:** Alexander Tacey, Tawar Qaradakhi, Tara Brennan-Speranza, Alan Hayes, Anthony Zulli, Itamar Levinger

**Affiliations:** 1 Institute for Health and Sport (IHES), Victoria University, Melbourne, VIC 3011, Australia; alexander.tacey@live.vu.edu.au (A.T.); tawar.qaradakhi@live.vu.edu.au (T.Q.); Alan.Hayes@live.vu.edu.au (A.H.); anthony.zulli@vu.edu.au (A.Z.); 2 Australian Institute for Musculoskeletal Science, Department of Medicine, Western Health, Melbourne Medical School, University of Melbourne, Melbourne, VIC 3021, Australia; 3 Department of Physiology and Bosch Institute for Medical Research, University of Sydney, Sydney, NSW 2006, Australia; tara.speranza@sydney.edu.au

**Keywords:** undercarboxylated osteocalcin, carboxylated osteocalcin, endothelial dysfunction, vascular calcification, atherosclerosis, humans, animal models

## Abstract

There is increasing evidence for the involvement of the skeleton in the regulation of atherosclerotic vascular disease. Osteocalcin, an osteoblast derived protein, exists in two forms, carboxylated and undercarboxylated osteocalcin. Undercarboxylated osteocalcin has been linked to the regulation of metabolic functions, including glucose and lipid metabolism. Features of atherosclerosis have been associated with circulating osteocalcin; however, this association is often conflicting and unclear. Therefore, the aim of this review is to examine the evidence for a role of osteocalcin in atherosclerosis development and progression, and in particular endothelial dysfunction and vascular calcification. The current literature suggests that undercarboxylated osteocalcin stimulates the phosphoinositide 3-kinase/protein kinase B (PI3K/Akt) signaling pathway to upregulate nitric oxide and nuclear factor kappa β (NF-кβ) in vascular cells, possibly protecting endothelial function and preventing atherogenesis. However, this effect may be mediated by metabolic factors, such as improvements in insulin signaling, rather than through a direct effect on the vasculature. Total osteocalcin is frequently associated with vascular calcification, an association that may occur as a result of vascular cells eliciting an osteogenic phenotype. Whether osteocalcin acts as a mediator or a marker of vascular calcification is currently unclear. As such, further studies that examine each form of osteocalcin are required to elucidate if it is a mediator of atherogenesis, and whether it functions independently of metabolic factors.

## 1. Introduction

Cardiovascular disease describes a group of disorders that affect the myocardium and vasculature and is the leading cause of death worldwide [1,2]. Ischemic heart disease, or atherosclerosis, is the most common cause of cardiovascular related deaths [3,4]. The development of atherosclerosis is characterized by distinct phases, including endothelial dysfunction, intimal thickening, plaque development, and, in the chronic stage, vascular calcification [5]. Numerous risk factors are responsible for the development of atherosclerosis, including diabetes, ageing, smoking, low physical activity levels, poor diet, and family history [6,7]. Diabetes, which is characterized by hyperglycemia and insulin resistance, is a leading risk factor for the development of atherosclerosis [2]. Abnormal insulin signaling, advanced glycation end-products, and oxidative stress caused by hyperglycemia and insulin resistance may promote the pathological interaction between diabetes and atherosclerosis [8,9,10,11].

Recently, the skeleton has been established as an endocrine organ participating in several metabolic processes, including the maintenance of circulating blood glucose and lipid levels. This bone/endocrine “cross talk” is thought to be regulated, at least in part, via osteocalcin, a vitamin K-dependent, osteoblast-derived protein [12,13,14,15]. Total osteocalcin includes both undercarboxylated osteocalcin (ucOC) and carboxylated osteocalcin (cOC). UcOC is characterized by the presence of 0–2 gamma-carboxyl groups on glutamic acid residues and is predominantly released into circulation [16]. The presence of 3 gamma-carboxyglutamic acid residues produces carboxylated osteocalcin (cOC), which has a high affinity for hydroxyapatite and is located predominantly in the bone matrix [17,18]. Vitamin K is essential for carboxylation and, as such, circulating levels of osteocalcin are used as a marker of vitamin K status [19]. Generally, ucOC makes up between 40 and 60% of total circulating osteocalcin [20,21,22]. The role of osteocalcin in the regulation of endocrine outcomes has been discussed in a number of recent review studies [23,24,25,26,27] and is thus not examined in this review.

The discovery of osteocalcin as a regulator of metabolic processes has led to investigations considering associations with cardiovascular disease. Several cross-sectional studies have demonstrated that circulating levels of total osteocalcin and ucOC are associated with both metabolic and cardiovascular disorders [28,29,30,31,32]. However, it is unclear whether osteocalcin has a direct role in the vasculature, independent of metabolic outcomes, or whether the association is mediated indirectly, via the metabolic effects of ucOC. Furthermore, advanced atherosclerotic plaques are characterized by the development of calcification, a process that involves a shift in vascular cells to an osteogenic phenotype [33]. Whether total osteocalcin, or one of the forms of osteocalcin, is a mediator or a marker of this process, is of interest. Osteocalcin has the potential to be a target in future therapeutic interventions for metabolic diseases such as diabetes and obesity [34]. Therefore, elucidating whether total osteocalcin and each of its forms have a biological function in the vasculature, independent from endocrine or metabolic outcomes, is of importance.

## 2. Atherosclerosis

Atherosclerosis typically presents in the coronary, cerebral, and peripheral arteries. The clinical manifestations are myocardial infarction, stroke, and peripheral arterial disease [35]. Each layer of the blood vessel contributes to the pathogenesis of atherosclerosis in a distinct way. For instance, the tunica intima, which is comprised primarily of a layer of endothelial cells, forms a semi-permeable barrier between the blood and the blood vessel wall [36]. Endothelial cell dysfunction is the initiating factor in atherogenesis and involves a reduction in the bioavailability of nitric oxide (NO), a major vasodilator and anti-atherogenic molecule. The reduction in NO results in an inflammatory response that is characterized by the migration of smooth muscle cells from the tunica media. The adherence of circulating molecules such as low-density lipoprotein (LDL) and cells, including leukocytes, contributes to the formation of a fatty streak [37]. Subsequently, a lipid rich plaque develops and collagen content increases. Finally, the advanced stages of atherosclerosis development are characterized by calcification and fibrosis [38]. Evidently, the phenotype of an atherosclerotic plaque alters dramatically over the life cycle of the disease. As such, it is of importance to distinguish between the stages when reporting atherosclerotic outcomes [39]. This review will focus on two distinct features of atherosclerosis, namely, the development of endothelial dysfunction and vascular calcification.

Endothelial dysfunction (vascular dysfunction) occurs in the initial stages of atherogenesis and is present throughout the life cycle of the disease. It is a predictor of future adverse cardiovascular events [40]. Normal endothelial function is regulated by a balance between anti-atherogenic (NO, endothelial derived hyperpolarizing factor, and prostacyclin) and pro-atherogenic (endothelin-1, thromboxane A2, and angiotensin II) factors [41,42]. The presence of a diseased state upsets the balance, suppressing atheroprotective factors and promoting atherogenic factors. This imbalance results in several pathological effects, principally the inability of the endothelium to regulate vasomotor tone (vasodilation and vasoconstriction) [36,43,44]. Abnormal vasomotor tone results in damage to the endothelial cells lining the vascular wall and is associated with an inflammatory and coagulatory response [43].

Advanced atherosclerosis, characterized by vascular calcification, involves the accumulation of scattered calcium-like deposits [38,45]. The presence of calcification reduces blood vessel compliance, causes stiffening of the vasculature, and is a predictor of future adverse health outcomes [46,47]. The development process of vascular calcification is similar to the process of bone formation in the skeleton [48]. This process involves the accumulation of hydroxyapatite, the differentiation of smooth muscle cells into osteoblast-like cells, the downregulation of calcification inhibitors, and the presence of osteogenic proteins, such as Runx-2 and osteocalcin [49,50,51].

## 3. Association between Osteocalcin and Atherosclerosis Outcomes

### 3.1. Measurement of Osteocalcin in Humans

To date, it is not clear whether osteocalcin is biologically active during atherosclerosis development in humans [16,27]. A recent meta-analysis included 46 studies examining the association between osteocalcin and atherosclerosis outcomes. No clear relationship was reported between osteocalcin and markers of atherosclerosis and calcification [52]. The presence of atherosclerosis was examined via several methods, including aortic calcification score (ACS), coronary artery calcification score, pulse wave velocity (PWV), and carotid intima-media thickness (C-IMT). Conflicting results were reported across all the outcomes. For example, in the studies reporting the association between osteocalcin and C-IMT, four reported that higher osteocalcin levels were associated with a higher C-IMT, four reported that higher osteocalcin levels were associated with a lower C-IMT, and three reported no correlation. Interestingly, all studies that examined osteocalcin-positive mononuclear cells or completed histological staining for osteocalcin reported that higher osteocalcin levels were associated with increased markers of atherosclerosis and calcification [52]. This suggests that osteocalcin may be present in atherosclerosis, although, whether it has a regulatory function is not clear.

The conflicting outcomes presented in the meta-analysis may be due to a number of limitations. First, total osteocalcin was reported in 43 of the studies, whereas some also included ucOC and cOC, reported in 7 studies and 1 study, respectively. This is a major limitation as each form has a distinct function [53,54]. Furthermore, different techniques were used to analyze osteocalcin. For example, total osteocalcin has been measured with enzyme-linked immunosorbent assay (ELISA) and radioimmunoassay (RIA), as well as flow cytometry and immunostaining, which may explain the different findings.

Taken together, the meta-analysis did not provide a clear association between osteocalcin and atherosclerosis. Consequently, the remainder of this review will focus on experimental studies that report the effect or expression of osteocalcin, specifically examining if each form of osteocalcin has distinct functions.

### 3.2. In Vivo Osteocalcin Treatment and Cardiovascular Function in Animal Models

The biological effects of osteocalcin are not fully known. A small number of studies have examined the effect of total osteocalcin administration in vivo on the cardiovascular system in animal models of disease. Tail cuff blood pressure (BP) was measured in apolipoprotein E-deficient (ApoE^−/−^) mice, and the animals received daily injections of total osteocalcin (30 ng/g) for 12 weeks [55]. Total osteocalcin treatment had a protective effect on high-fat-diet-induced hypertension by reducing mean and diastolic BP by ~5 and 7 mmHg, respectively. Similar trends occurred for systolic BP, which was decreased by ~3 mmHg. Although an improvement in blood pressure occurred, there was also a concomitant improvement in body weight, fasting blood glucose levels, glucose tolerance, circulating lipids, and markers of inflammation in animals receiving osteocalcin treatment [55]. As such, it is not clear whether the observed improvements were due to a direct effect of osteocalcin on the cardiovascular system, or indirectly via the improvement in the animals’ metabolic profile.

In another study, the administration of total osteocalcin repaired the alteration to PWV, concurrently with improvements in fasting blood glucose and circulating lipids [56]. Specifically, PWV, which is a measure of arterial stiffness, was increased (by 6%) in a rat model fed a high-fat diet and induced with diabetes via streptozotocin injection. Following 12 weeks of a high-fat diet, daily intraperitoneal injections of total osteocalcin (30 ng/g) reversed the alteration in PWV, albeit by a small magnitude. Blood pressure, heart rate, and mean arterial pressure were not altered by the high-fat diet or the osteocalcin injections [56]. This study suggests that a daily injection of total osteocalcin can repair abnormal cardiovascular function (Table 1). However, whether the protective effect occurred directly, or as a result of improved metabolic outcomes, is still not clear.

### 3.3. In Vivo Osteocalcin Treatment and Markers of Atherosclerosis Risk in Animal Models

Isometric/isotonic myography is an ex vivo technique used to examine endothelial function directly, independent of factors such as sheer stress and circulation hormones. This technique was used to examine the function of the thoracic aorta in ApoE^-/-^ mice following 12 weeks on a high-fat diet, receiving simultaneous daily injections of total osteocalcin (30 ng/g) or vehicle [55]. Vehicle-treated mice had a reduction in endothelial function by 20%, a pathological effect that was attenuated in the mice receiving osteocalcin treatment. An examination of the mechanisms revealed that co-incubation with N^G^-nitro-l-arginine methyl ester (L-NAME), an inhibitor of nitric oxide synthase, blocked the relaxation of all groups. However, co-incubation with sodium nitroprusside (SNP), a nitric oxide donor that is endothelium-independent, resulted in a similar relaxation between total osteocalcin-treated and non-treated tissue. The relaxation of all vessels to SNP demonstrates that the high-fat diet or total osteocalcin does not affect the ability of vascular smooth muscle cells to respond to nitric oxide [55]. Thus, total osteocalcin appears to have a protective effect on endothelial function that may assist in the prevention of atherosclerosis.

Whether one or both forms of osteocalcin were responsible for this effect is unclear. As a result, each form of osteocalcin was administered to female wild type C57BL/6 mice, to determine the effect on nitric oxide availability. Treatment with ucOC (30 ng/g), but not an equivalent dose of cOC, increased serum nitric oxide, providing further evidence that it is the bioactive form of the protein, at least in mice [57]. Subsequently, ucOC (30 ng/g) was administered via intraperitoneal injection 5 times per week for 10 weeks into mice fed an atherogenic (F2HFD1) diet. The diet did not induce the development of atherosclerotic plaques, but it did increase total cholesterol, LDL, and LDL/high density lipo-protein (HDL) ratio, all of which are associated with an increased risk of atherosclerosis. UcOC administration significantly lowered all the lipid markers and produced a 1.7-fold increase in serum nitric oxide bioavailability compared to saline-treated mice [57]. The improvement in lipid markers and serum nitric oxide availability would likely assist in the prevention of atherosclerosis development.

Furthermore, eight weeks of daily ucOC (30 ng/g) treatment following a high-fat diet produced an improvement in insulin signaling and a reduction in autophagy and ER stress in the aorta of C57BL/6J mice [58]. Several markers of autophagy (Atg7, p62, and light chain 3 II (LC3-II)) and ER stress (protein kinase-like endoplasmic reticulum kinase (PERK) and eukaryotic initiation factor 2α (eIF2α)) were increased in the high-fat diet-fed mice. However, the administration of ucOC following the high-fat diet attenuated the pathologic autophagy and ER stress marker response. Moreover, insulin resistance was detected in the high-fat-diet-fed mice, as measured by a reduction in the phosphorylation of insulin receptor β (IRβ) subunit tyrosine 1162/1163 and protein kinase B (Akt) Ser-473. An improvement in insulin signaling in ucOC-treated mice, as seen by an increase in the IRβ subunit and Akt Ser-473 phosphorylation, demonstrates that ucOC rescues high-fat-diet-induced insulin resistance in mouse aorta [58].

In summary, in vivo ucOC treatment protects vascular function and pathological disease markers that often contribute to or are involved in the development of atherosclerosis (Table 1). However, the protective effects of ucOC on the vasculature are often associated with improved metabolic outcomes, such as improvements in insulin signaling or lipid markers. As such, in vitro studies are needed to confirm (1) whether osteocalcin and its forms are acting directly on vascular tissue, and (2) that ucOC is the active form of osteocalcin mediating these effects.

## 4. Osteocalcin and Endothelial Function

### 4.1. In Vitro Osteocalcin Treatment in Human Cells

The endothelium has an important role in maintaining vascular homeostasis because it mediates the release of a number of regulatory factors [41]. Molecular signaling mechanisms that regulate vascular function have been examined in several studies to determine if there is a direct link between osteocalcin and atherosclerosis development (Table 2).

Human umbilical vein endothelial cells (HUVECs) cultured with total osteocalcin (10–150 ng/mL) displayed a dose-dependent upregulation of Akt and endothelial nitric oxide synthase (eNOS) phosphorylation—up to 100 ng/mL of total osteocalcin [55]. Akt is a common protein kinase involved in numerous cellular signaling pathways, including the phosphorylation of eNOS via serine1177; eNOS synthesizes NO [59]. When treated with 100 ng/mL of total osteocalcin, Akt and eNOS phosphorylation increased, peaking at 1 h and 2 h following treatment, respectively [55]. Of note, the properties of HUVECs are not ubiquitous to all endothelial cells, therefore the findings cannot be directly associated with adult endothelial function [60]. Despite this, similar results have been reported in human aortic endothelial cells (HAECs). HAECs incubated with ucOC or cOC for 30 min resulted in an increase in eNOS phosphorylation in cells treated with 25 and 100 ng/mL of ucOC by ~1-fold and ~2.5-fold, respectively. However, equivalent doses of cOC had no effect [57]. Similarly, eNOS phosphorylation and nitric oxide secretion were increased in a dose-dependent manner between 0.3–30 ng/mL of ucOC treatment in HAECs [61]. Mechanistic investigation demonstrated that the phosphorylation of Akt/eNOS by ucOC was inhibited by the addition of wortmannin, an inhibitor of phosphoinositide 3-kinase (PI3K), which is the protein kinase responsible for phosphorylating Akt [61].

**Figure 1:**
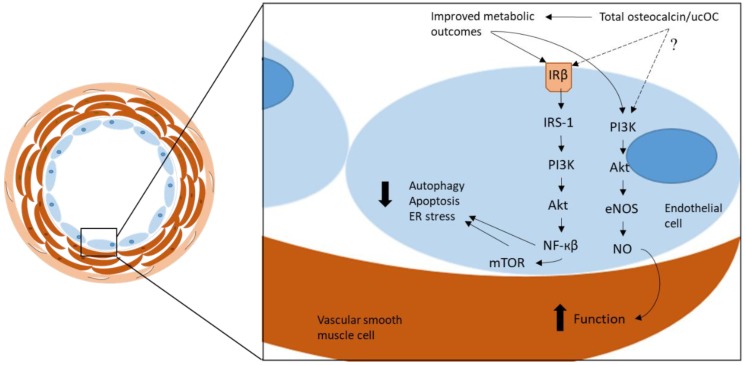
The proposed mechanism through which total osteocalcin/ucOC has been reported to elicit atheroprotective functions in vascular cells. By improving metabolic outcomes, total osteocalcin/ucOC reduces pathological mechanisms, including autophagy, apoptosis, and ER stress, through the β-subunit of the insulin receptor (IRβ) and via the IRS-1/PI3K/Akt/NF-кβ/mTOR signaling pathway. Vascular function is improved via the PI3K/Akt/eNOS signaling pathway which stimulates NO in the smooth muscle cells. ucOC = undercarboxylated osteocalcin, PI3K = phosphoinositide 3-kinase, Akt = protein kinase B, eNOS = endothelial nitric oxide synthase, NO = nitric oxide, IRβ = insulin receptor β, IRS-1 = insulin receptor substrate 1, NF-кβ = nuclear factor kappa β, mTOR = mammalian target of rapamycin, ER = endoplasmic reticulum.

Taken together, these results indicate that ucOC may upregulate nitric oxide synthesis via the activation of the PI3K/Akt/eNOS signaling pathway in human endothelial cells, which may have a protective effect against endothelial dysfunction. Again, these findings support previous research suggesting that ucOC is the biologically active form of the protein. UcOC may have a protective function in the vasculature, independent from its influence on metabolic outcomes, however further studies are required to confirm this (Figure 1).

Several pathological mechanisms promote the development of endothelial dysfunction and atherosclerosis, including elevated apoptosis and endoplasmic reticulum (ER) stress. UcOC (30 ng/mL) treatment prior to the administration of linoleic acid, which acts as a free fatty acid, inhibited the induction of apoptosis in HAECs via the PI3K/Akt pathway [61]. Additionally, HUVECs exhibited ER stress and insulin resistance when incubated with tunicamycin; however, co-incubation with ucOC (5 ng/mL) for 4 h reduced the ER stress and increased the phosphorylation of insulin receptor substrate 1 (IRS-1), a molecule involved in insulin signal transduction [62]. The co-incubation of wortmannin and Akti—1/2 (an Akt inhibitor) blocked the insulin sensitizing effect of ucOC. However, U0126 (a mitogen-activated protein kinase (MAPK) inhibitor) did not block the effect of ucOC. Additionally, nuclear factor kappa β (NF-кβ), a key cellular signaling molecule, which was suppressed by tunicamycin, exhibited a normalization when co-incubated with ucOC. Furthermore, the inhibition of NF-кβ signaling and the silencing of the NF-кβ p65 gene confirmed that NF-кβ was involved in the regulation of ER stress and insulin signaling by ucOC [62]. The results from this study suggest that ucOC suppresses ER stress via the PI3K/Akt/NF-кβ signaling pathway and that improved insulin sensitivity initiates this response.

Collectively, it appears that ucOC may elicit an atheroprotective effect in human endothelial cells. The protective effects of ucOC often occurred through improved insulin signaling or in the presence of high lipid content. However, ucOC also produced a protective effect in endothelial cells without the presence of any metabolic mediators, suggesting that osteocalcin may have a direct bioactive influence in human endothelial cells. 

### 4.2. In Vitro Osteocalcin Treatment and Markers of Atherosclerosis Risk in Animal Cells

Animal studies have also been used to examine the effect of osteocalcin in vascular tissue and cells. Experiments using cultured aortic strips obtained from ApoE^-/-^ mice revealed that total osteocalcin treatment increased the phosphorylation and expression of PI3K, Akt, and eNOS. Furthermore, the phosphorylation of Akt and eNOS was blocked by the co-incubation of LY294002 and Akt inhibitor V, which inhibit the signaling of PI3K and Akt, respectively [55]. UcOC (5 ng/mL) incubations in mouse vascular endothelial cells (VECs) and vascular smooth muscle cells (VSMCs) protected against tunicamycin-induced autophagy and ER stress. The protective effect was mediated through the Akt/mammalian target of rapamycin (mTOR) signaling pathway as a result of NF-кβ activation [58]. Similar results in cells from other organs revealed that these effects may be systemic. For example, ER stress and insulin resistance were both alleviated in L6 muscle cells when treated with 5 ng/mL of ucOC for 4 h [63].

Overall, total osteocalcin and ucOC appear to protect against the development of atherosclerosis through the activation of several signaling pathways (Table 2). NO is likely increased via the activation of the PI3K/Akt/eNOS signaling pathway, which would result in an improvement in endothelial function. However, increased eNOS expression can also enhance endothelial dysfunction by increasing eNOS uncoupling and oxidative stress [64,65].

Further studies are needed to determine if the upregulation of eNOS prevents or enhances endothelial dysfunction. Additionally, pathological abnormalities such as increased endothelial cell apoptosis and ER stress, which contribute to the development of atherosclerosis, are abrogated by ucOC treatment, likely acting through PI3K/Akt/NF-кβ/mTOR signaling. Whether these effects occur exclusively through metabolic signaling pathways, or if ucOC has a direct biological effect in the vasculature requires further investigation. 

## 5. Osteocalcin and Vascular Calcification

### 5.1. Osteocalcin and Calcified Human Vascular Tissue

Several osteogenic factors, including the osteoblastic “master regulator” transcription factor Runx2, have been shown to be expressed by smooth muscle cells located at the site of vascular calcified plaques [66]. The expression of such factors may play a role in the osteogenic potential of these smooth muscle cells. In addition, osteocalcin, which has been used clinically as a marker of bone formation, has also been found at these sites [67]. It has thus been posited that osteocalcin may play a role in the calcification of plaques, although whether this is true, or whether the association is coincidental, requires further investigation.

Advanced atherosclerotic plaques are characterized by arterial stiffening, resulting in a reduction in vessel compliance; this occurs as a consequence of vascular calcification [68]. Vascular calcification increases the risk of adverse cardiovascular events, including aortic stenosis, reduced vasomotor tone, and plaque instability [46]. Since the early 1980s, osteocalcin has been detected to a larger degree in calcified plaques and aortic valves than in non-calcified and healthy vessels [69,70]. In fact, in vessels obtained from men and women during autopsy, the concentration of total osteocalcin in calcified vascular plaques was considerably higher (50.9 ng/mL) than the concentration present in fatty streaks and fibrous plaques (1.1 ng/mL) and normal aortic tissue (0.33 ng/mL) [69]. Furthermore, a study a decade later reported that the level of osteocalcin mRNA was increased between 8- and 14-fold in calcified plaques and aorta compared to healthy aorta [71]. These findings predate the hypothesis that osteocalcin may have a role in vascular function, yet demonstrate that the concentration of total osteocalcin is positively correlated to the stage of atherosclerotic plaque progression. It is possible that the increase in total osteocalcin occurs as a result of atherosclerotic plaques developing an osteogenic phenotype [33], yet this requires further validation.

Vascular smooth muscle cells (VSMCs) are responsible for the development of atherosclerotic calcification by differentiating into osteoblast-like cells [50]. Cultured VSMCs in the initial stages of atherosclerosis formation undergo a downregulation of proteins that inhibit mineralization, leading to a shift in the VSMCs to an osteo/chondrocytic phenotype. This change is characterized by an increase in transcription factors regulating the expression of osteogenic proteins, including total osteocalcin [67]. Furthermore, total osteocalcin was shown to be minimally expressed in human aortic VSMCs cultured to mimic the early stages of atherosclerosis [72]. In situ hybridization analysis demonstrated that osteocalcin expression was significantly increased in lipid-rich, calcified VSMCs obtained from the media and intima of explanted human aorta, compared to VSMCs without plaque development [73]. Modification of lipid content by treating VSMCs for 28 days with acylated low-density lipoprotein did not alter osteocalcin expression, despite a 3-fold increase in calcification. Interestingly, incubation of lipoprotein-deficient serum for 28 days in VSMCs was associated with an inhibition of calcium formation, but an upregulation of osteocalcin expression [73]. This study demonstrates that VSMCs developing an osteogenic phenotype modify the expression of total osteocalcin and are influenced by the presence of lipids.

Overall, osteocalcin expression is increased in calcified lesions throughout the intimal and medial layers of the vascular wall (Table 3). Whilst the increase in osteocalcin is often associated with the development of an osteogenic phenotype, it is also associated with lipids. Of note, no studies have differentiated between the forms of osteocalcin when discussing vascular calcification. Consequently, an examination of ucOC and cOC and an investigation into the mechanisms that are associated with the increase in osteocalcin in calcified tissue are required.

### 5.2. Vascular Calcification and Osteocalcin in Animal Models

Vascular homeostasis is regulated by a tight balance between pro- and anti-mineralizing osteogenic factors. Several of these regulatory factors include matrix Gla protein (MGP), osteoprotegerin (OPG), fetuin A, and inorganic phosphate [79]. MGP and osteocalcin belong to the same family of mineral-binding Gla proteins, both characterized by vitamin K-dependent post translational y-carboxylation, and MGP is a known inhibitor of vascular calcification [80]. MGP-deficient mice are characterized by the development of calcified aortas and early death due to vascular thrombosis and hemorrhage [74,81]. MGP knockout mice were inter-crossed with pSM22α-Osteocalcin to create an osteocalcin gain-of-function model. Four-week-old pSM22α-Osteocalcin mice exhibited no difference in mineralization from the MGP knockout model, despite a 6- to 8-fold increase in serum osteocalcin concentration [74]. The results from this study demonstrate that total osteocalcin has no anti-mineralization effect.

Osteoprotegerin (OPG) is a circulating bone marker that functions to inhibit osteoclast activity [82]. OPG-deficient mice are characterized by osteoporosis [83]. Interestingly, OPG-deficient mice are associated with an increase in vascular calcification, suggesting that OPG may have a protective role in vascular calcification [75,83]. In a low-density lipoprotein receptor (ldlr) knockout mouse model treated with OPG and fed a high-fat diet, circulating total osteocalcin was reduced compared to vehicle-treated mice after two and five months. This was associated with the development of significantly less calcified lesions, suggesting that lower circulating total osteocalcin is correlated with a reduction in mouse aortic calcification [76]. Furthermore, cultured thoracic aorta of C57BL/6 mice revealed that increased calcification was positively correlated with an increase in osteocalcin expression [77]. As such, it appears that total osteocalcin is increased in animal tissue undergoing calcification, which may result from the differentiation of VSMCs to an osteogenic phenotype. Whether this association results in a biological effect, either protective or negative, cannot be determined from these studies.

A recent study reported that total osteocalcin stimulates glucose metabolism in vascular cells via hypoxia-inducible factor 1α (HIF-1α), a process that also results in the increase of vascular calcification [78]. Specifically, osteocalcin overexpression and treatment upregulated insulin signaling and the expression of glucose transporters through the increase in HIF-1α in mouse vascular smooth muscle cells (MOVAS). Furthermore, MOVAS cultured in DMEM medium for 21 days developed mineralized nodules and shifted to an osteogenic phenotype, a process that increases the expression of osteocalcin [78]. In an in vivo rat model of vascular calcification, total osteocalcin mRNA was positively associated with the presence of calcification. Immunohistochemistry analysis demonstrated similar expression patterns between total osteocalcin and the hypoxia-inducible factor 1α (HIF-1α) protein. Moreover, the silencing of osteocalcin RNA prevented HIF-1α stabilization and inhibited HIF-1α expression, which resulted in a reduction of calcification and a suppression of osteochondrogenic differentiation [78]. Altogether, this study demonstrates that total osteocalcin activates HIF-1α to upregulate glucose transport and utilization. The alteration to glucose metabolism stimulates osteogenic differentiation and promotes vascular calcification. 

To date, there are a limited number of studies that examine the biological effect of osteocalcin on vascular calcification. A number of different methodologies and models of calcification have been used, and importantly, no studies distinguished between each form of osteocalcin. Yet, most studies demonstrate that osteocalcin expression and concentration increase with the degree of calcification. However, the increase in total osteocalcin expression may be due to the fact that VSMCs differentiate into an osteogenic phenotype and produce osteocalcin. Further investigation is needed to determine if osteocalcin is a mediator or a marker of vascular calcification. 

## 6. The Putative Osteocalcin Receptor: GPRC6A

Osteocalcin’s receptor in the vasculature is yet to be identified. The G protein-coupled receptor family C, group 6, subtype A (GPRC6A) has been suggested as the putative receptor for osteocalcin in several tissues [84,85,86]. Previous studies have identified GPRC6A as the osteocalcin receptor in the testes [87], β-cells [88], and skeletal muscle [89,90] of mice. In humans, GPRC6A has been identified as the osteocalcin receptor in the testes [91,92]. However, several studies report that ucOC does not activate this G-protein [93,94].

There is minimal evidence regarding the role of GPRC6A as the receptor for osteocalcin in the vasculature. GPRC6A is present in the vasculature of rats [95], but whether osteocalcin interacts with this receptor is presently unknown. Overall, future studies should aim to elucidate whether GPRC6A is the receptor for osteocalcin in the vasculature, and also examine whether ucOC affects the MAPK pathway in human vascular cells.

## 7. Future Directions

The current review highlights that to date, most of the evidence for a link between osteocalcin and atherosclerosis and blood vessel function focused on total osteocalcin and, to a lesser extent, its forms (cOC and ucOC). Therefore, future studies should focus on the effect of each form of ucOC on atherosclerosis, blood vessels, and vascular calcification. Furthermore, it will be important to identify if there is a direct effect of ucOC on the vasculature or whether the interaction is indirect via an improvement in glucose regulation and glycemic control. As such, it is important to identify off-target effects.

## 8. Summary and Conclusions

The aim of this review was to examine the biological functions of osteocalcin and its forms throughout the atherosclerosis disease process and to determine whether it functions independently of metabolic outcomes. It appears that total osteocalcin and ucOC have the potential to improve endothelial function and reduce the pathological mechanisms that promote the development of atherosclerosis. This effect is often a result of improved metabolic outcomes; however, whether there is a direct osteocalcin/vascular interaction is yet to be fully elucidated. Further, the effect of osteocalcin on vascular calcification is unclear. In most cases, calcification was associated with the presence of total osteocalcin, which increased relative to the degree of calcification. However, whether it is a mediator or a marker of vascular calcification requires further investigation.

In conclusion, there is evidence to suggest that a cross-talk exists between the skeleton and the vascular system, which is associated with aspects of atherogenesis. Further research is required to determine whether total osteocalcin or each of its forms has a direct effect on the vasculature, independent from other systems.

## Figures and Tables

**Table 1:** Summary of studies examining the effects of in vivo osteocalcin treatment on vascular function outcomes in animals.

First Author, Year [Ref.]	Experimental Overview	Measurement of Vascular Function	Results
Dou, 2014 [55]	ApoE^-/-^ mice received ND or HFD and treatment daily for 12 weeks with vehicle or total osteocalcin (30 ng/g)	BP, heart rate, and isometric myography	In vivo: mean and diastolic BP normalized by osteocalcin treatment in HFD group, no change in systolic BP or heart rate. Ex vivo: 20% improvement in relaxation in osteocalcin-treated mice on HFD
Huang, 2017 [56]	Sprague Dawley rats induced with diabetes via STZ injection and received ND or HFD, daily treatment of vehicle or total osteocalcin (30 ng/g) for 12 weeks	BP, PWV, heart rate, pulse pressure, and mean arterial pressure	PWV normalized in osteocalcin-treated rats with diabetes compared to diabetic rats treated with vehicle, no change in BP, heart rate, mean arterial pressure, and pulse pressure
Kondo, 2016 [57]	Wild type C57BL/6 mice received HFD and treated 5 times a week for 10 weeks with vehicle or ucOC (30 ng/g)	Nitric oxide production	Increased nitric oxide concentration in ucOC-treated mice compared to vehicle-treated mice
Zhou, 2013 [58]	C57BL/6J mice received ND or HFD for 8 weeks with daily injections of vehicle or ucOC (30 ng/g)	Autophagy and ER stress	Autophagy and ER stress attenuated in mice receiving ucOC

ApoE = apolipoprotein E, HFD = high -fat diet, ND = normal chow diet, STZ = streptozotocin, ucOC = undercarboxylated osteocalcin, BP = blood pressure, PWV = pulse wave velocity, ER = endoplasmic reticulum.

**Table 2:** Summary of cell culture studies examining the effects of in vitro osteocalcin treatment in human and animal vascular cells.

First Author, Year [Ref.]	Experimental Overview	Outcomes	Results
Dou, 2014 [55]	HUVECs incubated with total osteocalcin (10–150 ng/mL) for 15 min–2 h. Descending aorta of ApoE^-/-^ mice, previously treated with osteocalcin, incubated with LY294002 (10 µmol/L) and Akt inhibitor V (5 µmol/L)	eNOS, Akt, and PI3K phosphorylation and expression	Max phosphorylation of eNOS and Akt with 100 ng/mL of osteocalcin. Max phosphorylation of eNOS and Akt occurred after 1 h and 2 h, respectively. In aorta, PI3K, Akt, and eNOS phosphorylation and expression increased, inhibited with LY294002 and Akt inhibitor V
Kondo, 2016 [57]	HAECs incubated with ucOC (5, 25, and 100 ng/mL) and cOC (25 and 100 ng/mL) for 30 min	eNOS phosphorylation	Incubation of ucOC increased eNOS phosphorylation in a dose-dependent manner, cOC had no effect
Jung, 2013 [61]	HAECs incubated with ucOC (0.3–30 ng/mL), linoleic acid (100 µmol/L for 16 h), and wortmannin (100 nmol/L for 15 min)	Nitric oxide concentration, eNOS and Akt phosphorylation and apoptosis	UcOC increased eNOS and Akt phosphorylation and nitric oxide concentration, which was inhibited by wortmannin. UcOC attenuated linoleic acid-induced apoptosis
Guo, 2017 [62]	HUVECs incubated with ucOC (5 ng/mL for 4 h), tunicamycin (5 µg/mL for 4 h), insulin (10 nM for 10 min), wortmannin, and Akti-1/2 (10 µM for 4 h)	Insulin resistance, ER stress	UcOC blocked ER stress and insulin resistance, which was inhibited by wortmannin and Akti-1/2
Zhou, 2013 [58]	Mouse VECs and VSMCs incubated with tunicamycin (5 µg/mL for 4 h), ucOC (5 ng/mL for 0, 2, 4, and 8 h), Akti-1/2 (10 µM for 4 h) and rapamycin (10 nM for 4 h)	Autophagy and ER stress	UcOC attenuated autophagy and ER stress in mouse VECs and VSMCs, which was inhibited by Akti-1/2 and rapamycin

HUVECs = human umbilical vein endothelial cells, ApoE = apolipoprotein E, HAECs = human aortic endothelial cells, ucOC = undercarboxylated osteocalcin, VECs = vascular endothelial cells, VSMCs = vascular smooth muscle cells, eNOS = endothelial nitric oxide synthase, Akt = protein kinase B, PI3K = phosphoinositide 3-kinase, ER = endoplasmic reticulum.

**Table 3:** Summary of studies examining the interaction between osteocalcin and calcification in human and animal tissue and cells.

First Author, Year [Ref.]	Experimental Overview	Outcomes	Results
Levy, 1983 [69]	Human aortic and valve tissue	Osteocalcin and Gla levels	Osteocalcin and Gla levels higher in calcified tissue than in non-calcified tissue
Levy, 1980 [70]	Human aortic and valve tissue	Gla levels	Higher Gla levels in calcified aorta and valves than non-calcified tissue
Fleet, 1994 [71]	Human aortic tissue	Osteocalcin mRNA levels	Osteocalcin mRNA increased in calcified aorta and plaque compared to non-calcified aorta
Tyson, 2003 [67]	Human aortic and carotid tissue	Osteocalcin expression	Calcified vessels had an increase in the expression of osteocalcin
Severson, 1995 [72]	Cultured human aortic VSMCs	Immunostaining for osteocalcin	Minimal immunostaining of human VSMCs
Proudfoot, 2002 [73]	Cultured human aortic VSMCs with lipid content modification	Osteocalcin expression	Osteocalcin expression increased in calcified cells compared to non-calcified cells, which was altered with the modification of lipid content
Murshed, 2004 [74]	MGP^-/-^ mice inter-crossed with pSM22α-Osteocalcin	Mineralization of aorta	Osteocalcin gain of function model did not inhibit the mineralization of mouse aorta
Pal, 2010 [75]	OPG ^+/+^ and OPG^-/-^ mice	Calcification and mononuclear cells expressing osteocalcin	Increased calcification in OPG^-/-^ mice, which was associated with an increased percentage of osteocalcin positive mononuclear cells
Morony, 2008 [76]	Ldlr ^-/-^ mice fed HFD for 5 months and treated with OPG	Calcification, osteocalcin mRNA and circulating levels	Osteocalcin mRNA levels were unchanged, circulating osteocalcin increased over the 5 months, which was associated with calcification
Akiyoshi, 2016 [77]	Thoracic aorta of C57BL/6 mice cultured to induced calcification	Osteocalcin expression	Osteocalcin expression increased in calcified thoracic aortas
Idelevich, 2011 [78]	Cultured MOVAS cells induced with calcification and overexpressed with osteocalcin. Sprague Dawley rats induced with calcification	Mineralization, osteocalcin mRNA, metabolic signaling pathways	In vitro: overexpression of osteocalcin in MOVAS cells associated with mineralization and upregulation of insulin signaling In vitro: osteocalcin mRNA is increased in calcified vasculature and associated with activation of metabolic signaling pathways

VSMCs = vascular smooth muscle cells, MGP = matrix Gla protein, OPG = osteoprotegerin, ldlr = low-density lipoprotein receptor, HFD = high-fat diet, MOVAS = mouse vascular smooth muscle cells.
